# In-hospital extracorporeal cardiopulmonary resuscitation for patients with out-of-hospital cardiac arrest: an analysis by time-dependent propensity score matching using a nationwide database in Japan

**DOI:** 10.1186/s13054-023-04732-y

**Published:** 2023-11-15

**Authors:** Yohei Okada, Sho Komukai, Taro Irisawa, Tomoki Yamada, Kazuhisa Yoshiya, Changhwi Park, Tetsuro Nishimura, Takuya Ishibe, Hitoshi Kobata, Takeyuki Kiguchi, Masafumi Kishimoto, Sung-Ho Kim, Yusuke Ito, Taku Sogabe, Takaya Morooka, Haruko Sakamoto, Keitaro Suzuki, Atsunori Onoe, Tasuku Matsuyama, Norihiro Nishioka, Satoshi Matsui, Satoshi Yoshimura, Shunsuke Kimata, Shunsuke Kawai, Yuto Makino, Kosuke Kiyohara, Ling Zha, Marcus Eng Hock Ong, Taku Iwami, Tetsuhisa Kitamura

**Affiliations:** 1 Department of Preventive Services, School of Public Health/Graduate School of Medicine, Kyoto University, Yoshida-Konoe-Cho, Sakyo, Kyoto 606-8501 Japan; 2 Health Services and Systems Research, Duke-NUS Medical School, National University of Singapore, Singapore, Singapore; 3 Division of Biomedical Statistics, Department of Integrated Medicine, Graduate School of Medicine, Osaka University, Suita, Japan; 4 Department of Traumatology and Acute Critical Medicine, Osaka University Graduate School of Medicine, Suita, Japan; 5 Emergency and Critical Care Medical Center, Osaka Police Hospital, Osaka, Japan; 6 Department of Emergency and Critical Care Medicine, Kansai Medical University, Takii Hospital, Moriguchi, Japan; 7 Department of Emergency Medicine, Tane General Hospital, Osaka, Japan; 8 Department of Critical Care Medicine, Osaka City University, Osaka, Japan; 9 Department of Emergency and Critical Care Medicine, Faculty of Medicine, Kindai University, Osaka-Sayama, Japan; 10 Osaka Mishima Emergency Critical Care Center, Takatsuki, Japan; 11 Critical Care and Trauma Center, Osaka General Medical Center, Osaka, Japan; 12 Osaka Prefectural Nakakawachi Medical Center of Acute Medicine, Higashi-Osaka, Japan; 13 Senshu Trauma and Critical Care Center, Osaka, Japan; 14 Senri Critical Care Medical Center, Saiseikai Senri Hospital, Suita, Japan; 15 Traumatology and Critical Care Medical Center, National Hospital Organization Osaka National Hospital, Osaka, Japan; 16 Emergency and Critical Care Medical Center, Osaka City General Hospital, Osaka, Japan; 17 Department of Pediatrics, Osaka Red Cross Hospital, Osaka, Japan; 18 Emergency and Critical Care Medical Center, Kishiwada Tokushukai Hospital, Osaka, Japan; 19 Department of Emergency and Critical Care Medicine, Kansai Medical University, Hirakata, Osaka Japan; 20 Department of Emergency Medicine, Kyoto Prefectural University of Medicine, Kyoto, Japan; 21 Division of Environmental Medicine and Population Sciences, Department of Social and Environmental Medicine, Graduate School of Medicine, Osaka University, Osaka, Japan; 22 Department of Food Science Faculty of Home Economics, Otsuma Women’s University, Tokyo, Japan; 23 Department of Emergency Medicine, Singapore General Hospital, Singapore, Singapore

**Keywords:** Extracorporeal cardiopulmonary resuscitation, Out-of-hospital cardiac arrest, Time-dependent propensity score matching, Nationwide database, Japanese, Rescue therapy

## Abstract

**Background:**

Extracorporeal cardiopulmonary resuscitation (ECPR) has been proposed as a rescue therapy for patients with refractory cardiac arrest. This study aimed to evaluate the association between ECPR and clinical outcomes among patients with out-of-hospital cardiac arrest (OHCA) using risk-set matching with a time-dependent propensity score.

**Methods:**

This was a secondary analysis of the JAAM-OHCA registry data, a nationwide multicenter prospective study of patients with OHCA, from June 2014 and December 2019, that included adults (≥ 18 years) with OHCA. Initial cardiac rhythm was classified as shockable and non-shockable. Patients who received ECPR were sequentially matched with the control, within the same time (minutes) based on time-dependent propensity scores calculated from potential confounders. The odds ratios with 95% confidence intervals (CI) for 30-day survival and 30-day favorable neurological outcomes were estimated for ECPR cases using a conditional logistic model.

**Results:**

Of 57,754 patients in the JAAM-OHCA registry, we selected 1826 patients with an initial shockable rhythm (treated with ECPR, *n* = 913 and control, *n* = 913) and a cohort of 740 patients with an initial non-shockable rhythm (treated with ECPR, *n* = 370 and control, *n* = 370). In these matched cohorts, the odds ratio for 30-day survival in the ECPR group was 1.76 [95%CI 1.38–2.25] for shockable rhythm and 5.37 [95%CI 2.53–11.43] for non-shockable rhythm, compared to controls. For favorable neurological outcomes, the odds ratio in the ECPR group was 1.11 [95%CI 0.82–1.49] for shockable rhythm and 4.25 [95%CI 1.43–12.63] for non-shockable rhythm, compared to controls.

**Conclusion:**

ECPR was associated with increased 30-day survival in patients with OHCA with initial shockable and even non-shockable rhythms. Further research is warranted to investigate the reproducibility of the results and who is the best candidate for ECPR.

**Graphical Abstract:**

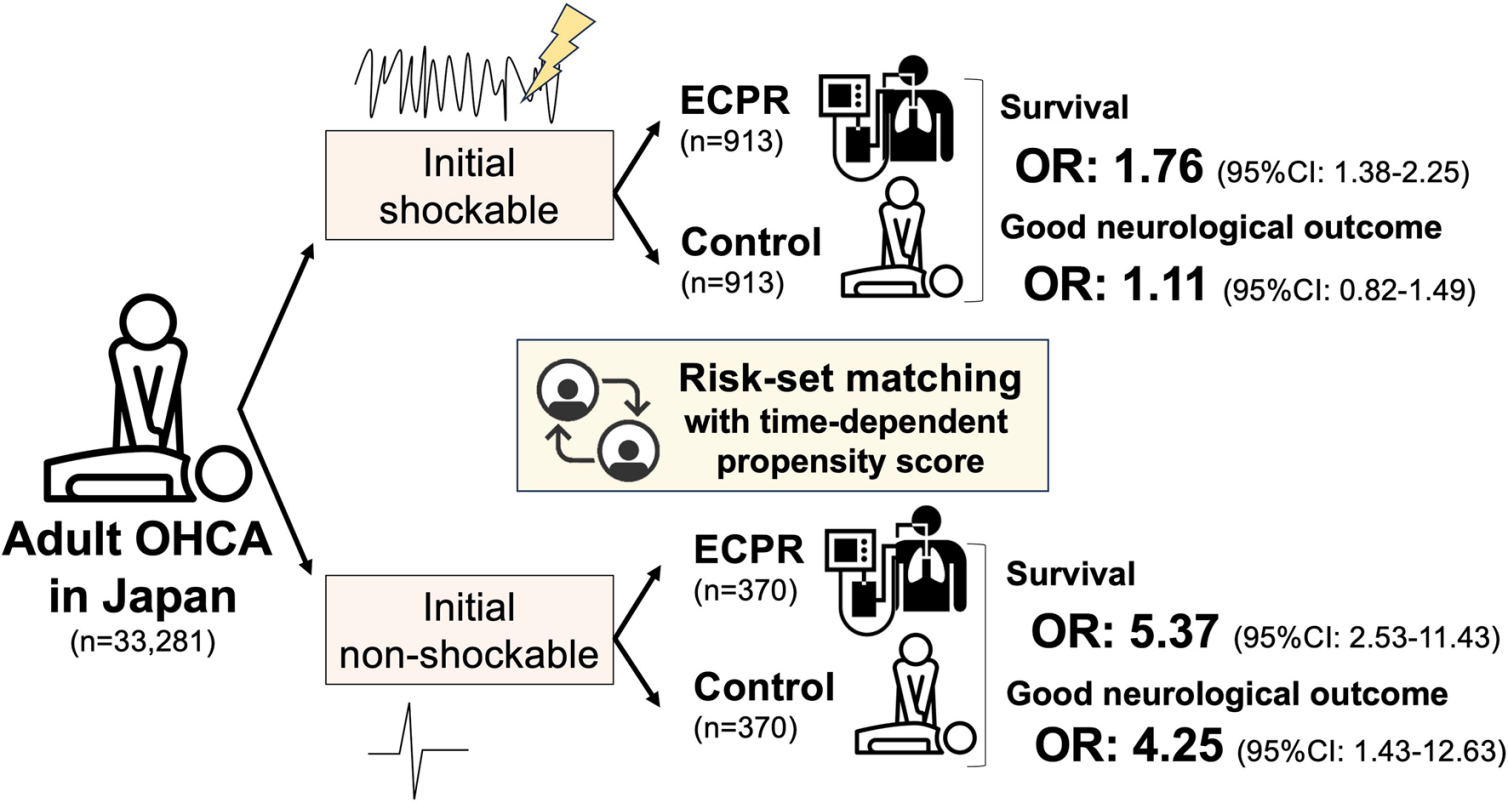

**Supplementary Information:**

The online version contains supplementary material available at 10.1186/s13054-023-04732-y.

## Background

Extracorporeal cardiopulmonary resuscitation (ECPR) is an advanced procedure used in addition to standard resuscitation in patients with refractory out-of-hospital cardiac arrest (OHCA). It involves the implementation of an extracorporeal circuit through emergent percutaneous cannulation of the femoral vein and artery to supply oxygenated blood and remove carbon dioxide from the brain and other vital organs [1, 2]. Currently, three randomized controlled trials (RCTs) and observational studies have been published, and their meta-analysis showed that ECPR potentially improves clinical outcomes of patients with OHCA; however, the effect on clinical outcomes and who is the best candidate of ECPR is still under discussion [3–11]. Hence, further investigation is needed to consider the effect of ECPR and who is the best candidate for ECPR.

While further research on the effects of ECPR is indeed necessary, conducting an RCT is challenging due to the practical burdens in clinical settings, costs, and ethical issues [12]. High-quality observational studies are therefore required to compensate for the limitations of conducting RCTs. However, prior observational studies on ECPR have raised concerns about the risk of bias, particularly with regard to the resuscitation time bias [10, 13]. To address this bias, some studies related to resuscitation used sequential risk-set matching with time-dependent propensity scores to evaluate interventions such as advanced airway management and adrenaline administration [14–16]. Previously, one study investigated the association between ECPR and outcomes using this time-dependent propensity score; however, this study did not divide the patients based on the initial cardiac rhythm, which is crucial for discussing the effectiveness of ECPR, because the patients with initial non-shockable rhythm have totally different clinical features, such as potential causes, resuscitation algorithm, risk factors, predictors and their outcomes among patients with OHCA treated with ECPR [17–22]. However, no studies have evaluated the association between ECPR and clinical outcomes considering these issues. This study aimed to investigate the association between ECPR and clinical outcomes among patients with OHCA using sequential risk-set matching analysis with a time-dependent propensity score to address resuscitation time bias.

## Methods

The study protocol and retrospective analysis was approved by the Ethics Committees of Kyoto University (approval ID: R1045), Osaka University (approval ID: 21304), and each participating institution, and the requirement for written informed consent was waived for this observational study.

### Data source and management

This study was a retrospective analysis of the JAAM-OHCA registry, a prospective, multicenter, nationwide database established by the steering committee of the Japanese Association of Acute Medicine. Details of the registry have been previously published [23, 24]. This database combines pre-hospital and in-hospital information and outcomes of patients with OHCA transported to the emergency departments of 93 institutions (69 university hospitals and/or tertiary critical care centers). Tertiary critical care centers are certified by the Ministry of Health, Labor, and Welfare in Japan and are required to provide highly specialized treatments such as ECPR, percutaneous coronary intervention, and intensive care 24 h a day. The other 24 hospitals were not critical care centers or university hospitals; they provided emergency medical services to the community. Pre-hospital information based on the standardized Utstein format was collected by paramedics [25] and verified by the Fire and Disaster Management Agency in Japan. In-hospital information, such as treatment in the emergency department and after admission to the ICU, was registered by clinicians or clinical data administrators using an electronic data capture system with a standardized reporting form [23, 24]. The JAAM-OHCA registry committee checked the logic and quality of the data, and anonymized data were provided to the researchers by the committee.

### Participants

This study included all consecutive patients with OHCA registered in the JAAM-OHCA registry between June 2014 and December 2019 based on the following inclusion criteria, adult with OHCA aged ≥ 18 years due to medical causes, resuscitation attempted by paramedics at the scene, and cardiac arrest sustained and confirmed upon hospital arrival. The exclusion criteria were opting out of the study, absence of a pre-hospital record, not receiving resuscitation attempts, age less than 18 years, cardiac arrest due to external causes (such as trauma, drowning, and choking), absence of cardiac arrest at the time of initial evaluation by paramedics, and ROSC before/on hospital arrival, which means that we excluded ECMO implementation for cardiogenic shock after ROSC. ROSC was defined as the presence of palpable pulses for more than 30 s [26]. Further, we also excluded those who were transferred to a hospital in which the ECPR cases were not registered in the database because those hospitals were assumed to lack the capacity to provide ECPR, those whose final disposition in the emergency department (ROSC, termination of resuscitation, or ECPR) was missing, and those whose time to final disposition, such as time from hospital arrival to termination of resuscitation, was zero. The study participants were stratified by initial cardiac rhythm into shockable and non-shockable groups as confirmed by paramedics at the scene, as the current international guidelines propose two different algorithms according to the initial rhythm [27, 28]. A shockable rhythm was defined as VF or pulseless ventricular tachycardia, and a non-shockable rhythm was defined as pulseless electrical activity or asystole.

### Exposure

ECPR is defined as emergency cannulation and veno-arterial extracorporeal membrane oxygenation for patients with OHCA who sustained cardiac arrest upon arrival at the hospital. The decision to perform ECPR was determined by the individual physicians attending to each patient. Generally, pre-hospital ECPR was not available during the study period in Japan.

### Outcome

The primary outcome was 30-day survival. The secondary outcome was 30-day survival with favorable neurological outcomes, defined as Cerebral Performance Category (CPC) 1 or 2. It is commonly used to evaluate the neurological status after OHCA as follows: category 1, good cerebral performance; category 2, moderate cerebral disability; category 3, severe cerebral disability; category 4, coma or vegetative state; and category 5, death/brain death [25]. The CPC was evaluated by clinicians or research assistants in each hospital and registered to the database.

### Data measurement, collection, and handling of missing data

The following clinical information was obtained from the database: sex, age, witnessed status, CPR performed by a bystander, pre-hospital initial cardiac rhythm, cardiac rhythm on hospital arrival, resuscitation time course, final disposition in the emergency department (ED), and outcome. Final disposition in the ED is defined as the situation on termination of chest compression which can be classified as ROSC, death (terminate the resuscitation effort), or the start of ECPR. The details of the other variables are provided in the Additional file (Additional file 1: Method S1). Missing data were imputed by a random forest-based imputation procedure using the “missForest” package for eligible study participants [29]. We describe the details of this imputation method in the Additional file (Additional file 1: Method S2).

### Statistical analysis

#### Patient and hospital characteristics

We described the patients’ characteristics and hospital information. Data are shown as median and interquartile range (IQR) for continuous variables and as numbers and percentages for categorical variables. We used standardized mean differences to describe the data in both the original and matched cohorts.

#### Time-dependent propensity scores and sequential risk-set matching

We performed time-dependent propensity score and risk-set matching analysis in each cohort of the initial cardiac rhythm to evaluate the association between ECPR and outcomes. The details of the time-dependent propensity scores and sequential risk-set matching are explained in the Additional file (Additional file 1: Method 3). To estimate the time-dependent propensity score of receiving ECPR after hospital arrival, we applied the fine-gray regression model in the presence of competing risk with time-dependent covariates [30]. In this model, we dealt with ROSC and death (termination of resuscitation) before ECPR as the competing risk. We also set 120 min after arrival at the hospital as censoring in the model. Time-dependent covariates were defibrillation, intubation, and adrenaline administration in the hospital. The time-independent covariates in the calculation of the propensity score were as follows: patient characteristics (sex and age), pre-hospital information (witness, bystander CPR, initial cardiac rhythm, defibrillation by bystander, physician-staffed ambulance or helicopter, defibrillation by paramedics, type of advanced airway, number of administrations of adrenaline, pre-hospital ROSC, and time from call to hospital), and hospital capability of ECPR. Hospital capability for ECPR was defined as “high,” “middle,” or “low,” according to the tertiles of the number of patients who received ECPR in the last two years. Although we adopted the same variables to calculate the time-dependent propensity score for both shockable and non-shockable cohorts, the scores were calculated separately for each rhythm cohort.

Based on the time-dependent propensity scores, we performed 1:1 sequential matching of the patients who received ECPR at any given minute from minute 0 to minute 120 (patients treated with ECPR) to a patient who was at risk of receiving ECPR within the same minute (control) with replacement. In this sequential matching, at-risk patients (controls) included those who were still undergoing resuscitation at the emergency department and had not received ECPR before or within the same minute. Matching with replacements of unexposed patients meant that matched controls with no ECPR at each time were allowed to match again later until they received ECPR. Therefore, at-risk patients also included those who received ECPR later, as matching was not dependent on future events. In sequential matching, we set the caliper width for nearest-neighbor matching at 0.2 standard deviations of the propensity score, as recommended in previous literature [31]. We calculated standardized differences to evaluate the balance of variables in each propensity score-matched cohort. We considered a standardized mean difference of < 0.25, as suggested in the literature [31].

#### Estimation of association between ECPR and outcomes

To evaluate the association between ECPR and each outcome, we fitted a conditional logistic regression model to estimate odds ratios (ORs) with 95% confidence intervals (CIs) to compare to those at risk of receiving ECPR (control). All tests were two-sided; we regarded *p* < 0.05 as statistically significant. Data analysis was conducted from March 2022 to July 2022. All statistical analyses were performed using R software, version 4.1.2 (R Foundation for Statistical Computing).

## Results

### Study flowchart and original cohort

**Fig. 1:**
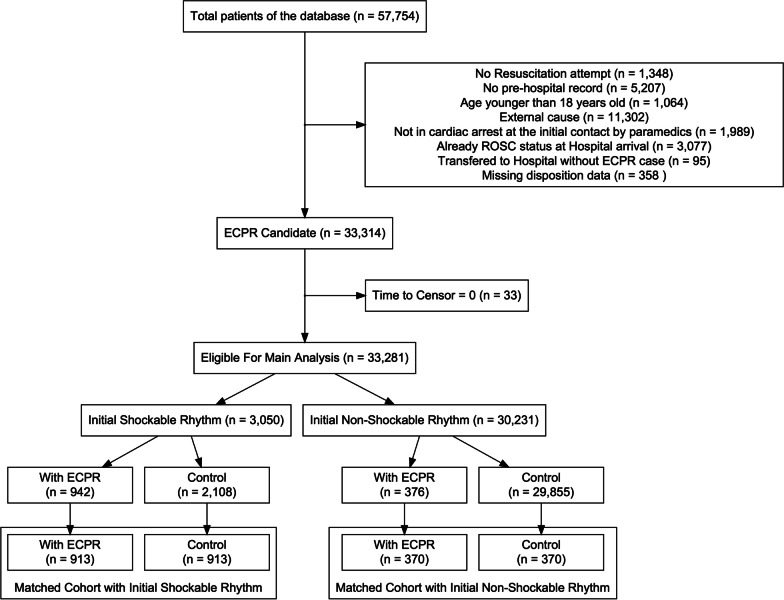
Study flowchart. ECPR, Extracorporeal cardiopulmonary resuscitation, ROSC, Return of spontaneous circulation

Of the 57,754 patients registered in the JAAM-OHCA registry, 3,050 patients with OHCA with initial shockable rhythm (942 patients treated with ECPR and 2,108 cases without ECPR) and 30,231 patients with OHCA with initial non-shockable rhythm (376 cases treated with ECPR and 29,855 cases without ECPR) were included in the analysis (Fig. 1). Details of the missing variables are described in the Additional file (Additional file 1: Table S1). The characteristics before matching are described in Table 1 and the Additional file (Additional file 1: Table S2).

**Table 1:** Patient characteristics in the original cohort

	Initial shockable rhythm			Initial non-shockable rhythm		
Characteristics	ECPR (*N* = 942)	Without ECPR (*N* = 2,108)	SMD	ECPR (*N* = 376)	Without ECPR (*N* = 29,855)	SMD
Sex (men)	811 (86%)	1,652 (78%)	0.20	289 (77%)	17,167 (58%)	0.42
Age	59 (48, 68)	69 (58, 79)	-0.68	60 (47, 69)	79 (68, 86)	-1.2
Witnessed	738 (78%)	1,465 (69%)	0.20	290 (77%)	11,232 (38%)	0.87
Bystander CPR	522 (55%)	1,076 (51%)	0.09	164 (44%)	14,350 (48%)	-0.09
Initial cardiac rhythm			0.00			0.80
Initial VF/VT	942 (100%)	2,108 (100%)		-	-	
PEA	-	-		241 (64%)	8,141 (27%)	
Asystole	-	-		135 (36%)	21,714 (73%)	
Bystander AED	79 (8.4%)	114 (5.4%)	0.12	22 (5.9%)	260 (0.9%)	0.28
Physician-staffed heli/ambulance	249 (26%)	383 (18%)	0.20	87 (23%)	2,723 (9.1%)	0.39
Shock by paramedic	936 (99%)	2,046 (97%)	0.17	135 (36%)	1,315 (4.4%)	0.85
Advanced airway			0.12			0.03
Intubation	122 (13%)	201 (9.5%)		36 (9.6%)	3,084 (10%)	
None	364 (39%)	905 (43%)		153 (41%)	12,361 (41%)	
SGA	456 (48%)	1,002 (48%)		187 (50%)	14,410 (48%)	
Number of IV adrenaline			0.10			0.17
0	554 (59%)	1,356 (64%)		231 (61%)	21,534 (72%)	
1	123 (13%)	255 (12%)		38 (10%)	2,474 (8.3%)	
2	102 (11%)	197 (9.3%)		45 (12%)	2,094 (7.0%)	
3	91 (9.7%)	157 (7.4%)		36 (9.6%)	1,943 (6.5%)	
4	36 (3.8%)	73 (3.5%)		21 (5.6%)	927 (3.1%)	
5	36 (3.8%)	70 (3.3%)		5 (1.3%)	883 (3.0%)	
Pre-hospital ROSC	73 (7.7%)	238 (11%)	-0.12	30 (8.0%)	1,048 (3.5%)	0.19
Time from call to hospital	31 (26, 39)	32 (26, 40)	-0.08	33 (27, 40)	33 (27, 40)	0.00
Initial cardiac rhythm on arrival			0.72			1.1
Asystole	154 (16%)	770 (37%)		111 (30%)	22,622 (76%)	
PEA	167 (18%)	658 (31%)		172 (46%)	6,776 (23%)	
VF/VT	621 (66%)	680 (32%)		93 (25%)	457 (1.5%)	
Shock in hospital	724 (77%)	991 (47%)	0.65	200 (53%)	2,014 (6.7%)	1.2
Adrenaline in hospital	855 (91%)	1,915 (91%)	0.00	351 (93%)	26,191 (88%)	0.19
Intubation in hospital	809 (86%)	1,679 (80%)	0.17	334 (89%)	19,236 (64%)	0.60
ECPR case volume			0.51			0.55
High volume	688 (73%)	1,122 (53%)		274 (73%)	15,765 (53%)	
Middle volume	209 (22%)	579 (27%)		85 (23%)	7,874 (26%)	
Low volume	45 (4.8%)	407 (19%)		17 (4.5%)	6,216 (21%)	

Continuous variables are expressed as medians and interquartile ranges, and categorical variables are expressed as number and percentage (%)

SMD, standardized mean difference; ECPR, extracorporeal cardiopulmonary resuscitation; CPR, cardiopulmonary resuscitation; VF, ventricular fibrillation; VT, ventricular tachycardia; PEA: pulseless electrical activity; ROSC, return of spontaneous circulation; AED, automated external defibrillator; SGA, supraglottic airway; IV, intravenous

### Matched cohort

Through matching using a time-dependent propensity score, 1,826 initially shockable cases (913 cases treated with ECPR and 913 controls) and 740 initially non-shockable cases (370 cases treated with ECPR and 370 controls) were included in the primary analysis. In the matched cohort with initial shockable rhythm, ECPR versus control, median (IQR) ages were 59 [48–68] and 59 [48–70] years and males were 86% (784/913) and 85% (778/913), respectively. In the non-shockable matched cohort, ECPR versus control, median (IQR) ages were 60 [47–69] and 58 [45–69] years and males were 77% (284/370) and 75% (278/370), respectively. In the ECPR group, the median (IQR) time to ECPR initiation was 25 [6, 18] minutes in patients with initial shockable rhythm and 28 [3, 20] minutes in patients with initial non-shockable rhythm. Among the control groups, ECPR was performed in 389 (43%) and 60 (16%) patients in the initial shockable and non-shockable groups, respectively. Additional details of the characteristics are described in Table 2 and the Additional file (Additional file 1: Tables S3-S5). Overall, patient characteristics were well-balanced.

**Table 2:** Patient characteristics in the matched cohort

	Initial shockable rhythm			Initial non-shockable rhythm		
Characteristics	ECPR (*N* = 913)	Control (*N* = 913)	SMD	ECPR (*N* = 370)	Control (*N* = 370)	SMD
Sex (men)	784 (86%)	778 (85%)	0.02	284 (77%)	278 (75%)	0.04
Age	59 (48, 68)	59 (48, 70)	− 0.04	60 (47, 69)	58 (45, 69)	0.08
Witnessed	714 (78%)	717 (79%)	− 0.01	285 (77%)	296 (80%)	− 0.07
Bystander CPR	506 (55%)	510 (56%)	− 0.01	160 (43%)	178 (48%)	− 0.10
Initial cardiac rhythm			0.00			0.01
Initial VF/VT	913 (100%)	913 (100%)		–	–	
PEA	–	–	–	237 (64%)	239 (65%)	
Asystole	–	–	–	133 (36%)	131 (35%)	
Bystander AED	77 (8.4%)	74 (8.1%)	0.01	20 (5.4%)	20 (5.4%)	0.00
Physician-staffed heli/ ambulance	237 (26%)	246 (27%)	− 0.02	86 (23%)	105 (28%)	− 0.12
Shock by paramedic	907 (99%)	896 (98%)	0.11	133 (36%)	114 (31%)	0.11
Advanced airway			0.08			0.09
Intubation	121 (13%)	126 (14%)		35 (9.5%)	41 (11%)	
None	349 (38%)	379 (42%)		151 (41%)	160 (43%)	
SGA	443 (49%)	408 (45%)		184 (50%)	169 (46%)	
Number of IV adrenaline			0.02			− 0.05
0	533 (58%)	538 (59%)		226 (61%)	212 (57%)	
1	121 (13%)	135 (15%)		38 (10%)	56 (15%)	
2	99 (11%)	93 (10%)		44 (12%)	48 (13%)	
3	91 (10.0%)	72 (7.9%)		36 (9.7%)	22 (5.9%)	
4	33 (3.6%)	34 (3.7%)		21 (5.7%)	10 (2.7%)	
≥ 5	36 (3.9%)	41 (4.5%)		5 (1.4%)	22 (5.9%)	
Pre-hospital ROSC	70 (7.7%)	67 (7.3%)	0.01	30 (8.1%)	25 (6.8%)	0.05
Time from call to hospital	31 (26, 39)	31 (25, 38)	0.06	33 (27, 40)	34 (27, 41)	− 0.03
Initial cardiac rhythm on arrival			0.09			0.11
Asystole	151 (17%)	133 (15%)		110 (30%)	104 (28%)	
PEA	162 (18%)	143 (16%)		169 (46%)	188 (51%)	
VF/VT	600 (66%)	637 (70%)		91 (25%)	78 (21%)	
Shock in hospital	472 (52%)	551 (60%)	− 0.17	133 (36%)	133 (36%)	0.00
Adrenaline in hospital	776 (85%)	838 (92%)	− 0.21	332 (90%)	346 (94%)	− 0.14
Intubation in hospital	711 (78%)	761 (83%)	− 0.14	303 (82%)	323 (87%)	− 0.15
ECPR case volume			0.12			0.19
High volume	668 (73%)	664 (73%)		269 (73%)	277 (75%)	
Middle volume	202 (22%)	181 (20%)		85 (23%)	64 (17%)	
Low volume	43 (4.7%)	68 (7.4%)		16 (4.3%)	29 (7.8%)	

Continuous variables are expressed as medians and interquartile ranges, and categorical variables are expressed as numbers and percentages (%)

SMD, standardized mean difference. ECPR, Extracorporeal cardiopulmonary resuscitation; CPR, Cardiopulmonary resuscitation; VF, Ventricular fibrillation; VT, Ventricular tachycardia; PEA: Pulseless electrical activity; ROSC, Return of spontaneous circulation; AED, Automated external defibrillator; SGA, Supraglottic airway; IV, intravenous. Variables were included in the model to generate propensity scores

### Outcomes in the initially shockable rhythm matched cohort

**Fig. 2:**
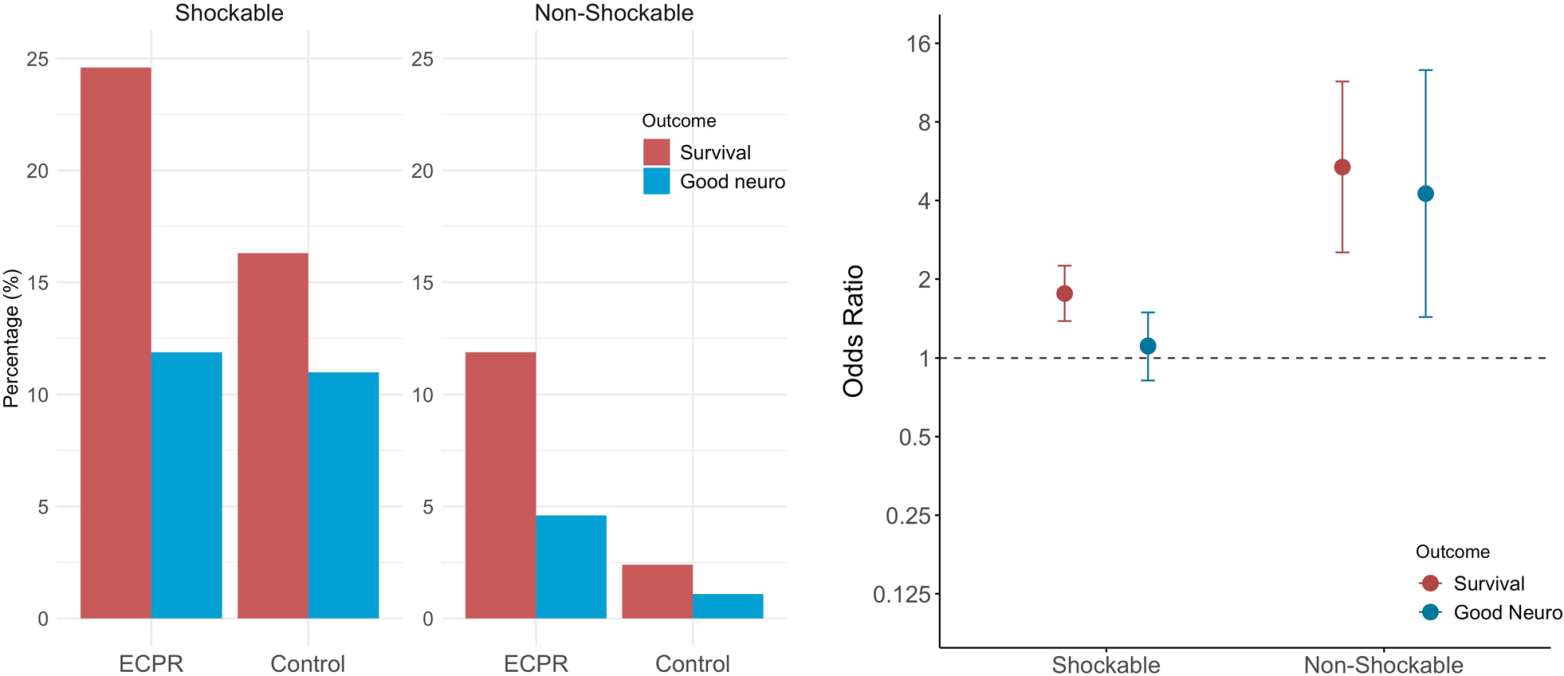
Main results of the matched cohorts. ECPR, Extracorporeal cardiopulmonary resuscitation, Survival, 30-day survival, Good Neuro, 30-day good neurological outcome. Left: Percentage of 30-day survival and good neurological outcome in the matched cohorts (initial shockable and initial non-shockable rhythm). Right: Odds ratio of the ECPR group for 30-day survival and good neurological outcome in the matched cohorts (initial shockable and initial non-shockable rhythm)

The 30-day survival rate in the cohort with initial shockable rhythm was 24.6% (225/913) in the ECPR group and 16.3% (149/913) in the control group (Fig. 2). The OR by the conditional logistic regression model for 30-day survival with ECPR was 1.76 [95%CI 1.38–2.25] compared with the control. However, in the matched cohort with initial shockable rhythm, 30-day favorable neurological outcomes accounted for 11.9% (109/913) in the ECPR group and 11.0% (100/913) in the control group. The OR for 30-day favorable neurological outcome was 1.11 [95%CI 0.82–1.49] for ECPR compared with the control (Fig. 2).

### Outcomes in the initially non-shockable rhythm matched cohort

The 30-day survival rate in the matched cohort with initial non-shockable rhythm was 11.9% (44/370) for the ECPR group and 2.4% (9/370) for controls. The OR for 30-day survival was 5.37 [95%CI 2.53–11.43] for ECPR compared with the control (Fig. 2). The 30-day favorable neurological outcomes in the matched cohort with initial non-shockable rhythm accounted for 4.6% (17/370) in the ECPR group and 1.1% (4/370) in the control group. The OR for 30-day favorable neurological outcome was 4.25 [95%CI 1.43–12.63] for ECPR compared with the control (Fig. 2).

## Discussion

### Key observations and strengths

This observational study using risk-set matching by time-dependent propensity score indicated that ECPR was associated with higher survival rates among adult patients with OHCA with both initial shockable and non-shockable rhythms. Furthermore, ECPR was associated with favorable neurological outcomes in patients with OHCA with non-shockable rhythm. Although some previous observational studies investigated the association between ECPR and clinical outcomes, they did not adequately address resuscitation time bias and time-dependent confounding [10]. The strength of this study is to show the association between ECPR and outcomes using the robust methodology to address resuscitation time bias among patients with OHCA with and without initial shockable rhythm.

### Interpretation and clinical implications

ECPR was associated with improved survival and favorable neurological outcomes among patients with OHCA with an initial non-shockable rhythm. The potential mechanism explaining this finding may be that ECPR allows time to address the treatable causes of non-shockable rhythms, such as dialysis for hyperkalemia or anticoagulation therapy for pulmonary embolization, which may improve outcomes in selected patients with OHCA with initial non-shockable rhythms [19]. Accordingly, the appropriate selection of ECPR candidates can lead to better outcomes even the patients with OHCA initially had non-shockable rhythms.

In contrast, although patients with OHCA with shockable rhythm are recognized as the target population for ECPR, this study indicated that ECPR was not associated with favorable neurological outcomes among patients with OHCA with an initial shockable rhythm. We guess the reason that ECPR was administered to some of these patients even if their brains had already been hypoxically injured. These patients may have had better survival with ECPR but not improved neurological outcomes. In Japan, ECPR is commonly implemented according to the criteria suggested by the SAVE-J study; [7] however, the final decision of using ECPR is made by the physician in charge of the patient, and the indications for ECPR vary between institutions [32]. This study included patients from a wide range of institutions and time periods, including cases where the time from call to hospital arrival was longer than 40 min or the time from hospital arrival to start of ECPR was more than 60 min. Worse neurological outcomes have been reported with longer durations to ECPR initiation [21]. We opine that some of the patients received ECPR after the “golden period”; hence, the effect of ECPR might has been diluted. The RCT recently published investigating the effect of ECPR among patients with OHCA with an initial shockable rhythm also did not show the favorable effect of ECPR on the neurological outcome, and in this trial, the duration between the emergency call and the start of ECPR in most cases was more than 60 min (median 74 [IQR: 63–87] minutes) [5]. We did not analyze the time to hospital or time to start ECPR because of the limited sample size; however, future studies should investigate the heterogeneity of the effect of ECPR based on the time to determine the optimal timing for ECPR.

However, we believe that it is not appropriate to conclude that ECPR is not suitable for patients with OHCA with an initial shockable rhythm based on our results that ECPR was not associated with good neurological outcomes. A previous RCT (the ARREST trial) and meta-analysis of some RCTs had already indicated a favorable effect of ECPR on neurological outcomes of selected OHCA patients with initial shockable rhythm [3, 6, 33]. Rather, the results in this study suggested that all the patients with OHCA with an initially shockable rhythm treated with ECPR in this database may not be the best target population for ECPR in terms of neurological outcomes and it might be better to reconsider the current criteria among the institutions included in this database. We previously developed a clinical score for predicting the probability of favorable outcomes among patients with OHCA with a shockable rhythm treated with ECPR [34, 35] based on the following criteria: age less than 65 years, shockable rhythm at hospital arrival, time from call to hospital shorter than 25 min, and pH higher than 7.0 in the initial blood gas assessment. If the score is more than three, the probability of a favorable neurological outcome is predicted as approximately 20–30%. Therefore, ECPR may be more effective in the selected population. Here, we were unable to perform a subgroup analysis to investigate the population that may benefit from ECPR because risk-set matching would have been disturbed if the study population was divided by these factors. However, further observational research is needed to suggest the appropriate patients with OHCA for ECPR to inform further RCTs.

## Limitations

This study has some limitations: first, it has a potential risk of selection bias. The indication to perform ECPR was decided not based on unified criteria but on each clinician’s judgment. Most patients in this study were reasonable candidates for ECPR based on their characteristics; however, imperfect inclusion criteria may lead to concerns about generalizability. The estimated association was the average treatment effect on the treatment group, and the results may be influenced by patient selection. Second, the JAAM-OHCA database did not include complete clinical details, such as the clinical course after admission or details of the ECPR procedures. Third, although we addressed potential confounding factors, there may be a risk of unmeasured confounding factors, such as patients’ comorbidities or the quality of care in each facility. Fourth, since the control group was a combination of patients who never had undergone ECPR or who had ECPR performed later than the ECPR group, the effect estimate would more likely be closer to null (dilution of the effect) compared to that of a randomized trial that would strictly compare ECPR treatment policy to no ECPR. This corresponds to an intention-to-treat analysis with several protocol deviations that patients end up having ECPR later in the “no ECPR” group. In the initial shockable group, the magnitude of this dilution effect might be larger because the proportion of patients who underwent ECPR later in the control group was high (43%). Furthermore, favorable neurological outcomes in the control group were relatively high (11%) compared to those in a previous study in Japan (SAVE-J study), where it was approximately 2% among patients with OHCA who had an initial shockable rhythm, did not obtain ROSC, and ECPR was not performed [36]. Dilution of effect might be caused by sequential matching. Fifth, although evaluating CPC was reported to have substantial inter-rater reliability, there might be a risk of bias of misclassification in outcome assessment [4]. Sixth, we set survival as the primary outcome of this study, when planning our analysis, we observed that several pivotal studies on ECPR mainly considered survival as their primary outcome [3, 37]. Nonetheless, from a patient’s perspective, neurological outcomes hold greater significance. Therefore, we recommend a cautious interpretation and application of our findings. Seventh, this study was not conducted based on the pre-registered analysis plan or protocol; thus, this study may be assessed as having some risk of bias, especially in the domain of selection of the reported results [38]. Finally, although this study included study participants from nationwide data in Japan, there are some concerns about generalizability to other settings such as countries that have different emergency medical systems. The reproducibility of the results should be confirmed in other settings to address these issues.

## Conclusion

Although there are several limitations, this observational study using risk-set matching by time-dependent propensity score suggested that ECPR was associated with a higher 30-day survival among patients with OHCA with both initial shockable and even non-shockable rhythms. Furthermore, this study indicated ECPR was also associated with favorable neurological outcomes among patients with OHCA with initial non-shockable rhythms; however, not among patients with OHCA with initial shockable. Further research is warranted to investigate the reproducibility of the results and who is the best candidate for ECPR.

### Supplementary Information

**Additional file 1:** Supplemental Methods 1–3. Tables S1–S5.

## Data Availability

The datasets and/or analyses in this study are not publicly available because the ethics committee did not permit them.

## Abbreviations

ECPR: Extracorporeal cardiopulmonary resuscitation
OHCA: Out-of-hospital cardiac arrest
RCTs: Randomized controlled trials
VF: Ventricular fibrillation
ROSC: Return of spontaneous circulation
CPC: Cerebral Performance Category
IQR: Interquartile range
ORs: Odds ratios
CIs: Confidence intervals
