# Supplementary material for: In-hospital extracorporeal cardiopulmonary resuscitation for patients with out-of-hospital cardiac arrest: an analysis by time-dependent propensity score matching using a nationwide database in Japan
Source: Crit Care. 2023 Nov 15;27:442. doi: 10.1186/s13054-023-04732-y (PMC10652510; doi:10.1186/s13054-023-04732-y)
Supplement: Supplementary file 1 — Additional file 1: Supplemental Methods 1–3. Tables S1–S5. [file 13054_2023_4732_MOESM1_ESM.docx]

**Supplementary file**

**Title:**

In-Hospital Extracorporeal Cardiopulmonary Resuscitation for Patients With Out-Of-Hospital Cardiac Arrest: An Analysis By Time-Dependent Propensity Score Matching Using A Nationwide Database In Japan

**Contents**

| **S-Method 1.** | **Description about JAAM-OHCA database** |
| --- | --- |
| **S-Method 2.** | **Missing imputation** |
| **S-Method 3.** | **Additional explanation for method** |
| **S-Table 1.** | **Missingness of the variables** |
| **S-Table 2.** | **Disposition in the original cohort** |
| **S-Table 3.** | **Disposition in the matched cohort** |
| **S-Table 4.** | **Time of ECPR before matching** |
| **S-Table 5.** | **Time of ECPR after matching** |

**S-Method 1. Description of the variables in the JAAM-OHCA database**

| Variable | Description | Included in the model* |
| --- | --- | --- |
| Basic demographics | |  |
| Sex | Sex (Men/Women) | Fixed |
| Age | Age (years) | Fixed |
| Prehospital information (Ustein style) | |  |
| Witnessed | Witness of collapse (yes/no) | Fixed |
| Bystander CPR | Bystander CPR (yes/no) | Fixed |
| Initial cardiac rhythm | The cardiac rhythm initially confirmed by paramedics at the scene (VF or VT/PEA/Asystole) | Fixed |
| Bystander AED | Defibrillation performed by Bystander (yes/no) | Fixed |
| Physician Staffed Heli/Ambulance | Resuscitation performed by the physician of physician-staffed helicopter or ambulance (yes/no) | Fixed |
| Shock by Paramedic | Defibrillation performed by EMS paramedics (yes/no) | Fixed |
| Advanced Airway | Advanced airway management performed by paramedics (Intubation/Supraglottic airway/None) | Fixed |
| Number of IV adrenaline | Number of administrations of adrenaline via iv route by paramedics (0/1/2/3/4/5 or more) | Fixed |
| Prehospital ROSC | ROSC at the scene or during the transportation (yes/no) | Fixed |
| Time from Call to Hospital | The time from emergency call to the patients arrived at hospital (minute) | Fixed |
| In-hospital information | |  |
| Initial Cardiac Rhythm on Arrival | Initial cardiac rhythm confirmed on hospital arrival (VF or VT/PEA/Asystole) | Fixed |
| Shock in Hospital | The defibrillation in emergency department (yes/no) | Time-dependent |
| Time to shock from Arrival | Time to perform the defibrillation from hospital arrival in emergency department (minute) |  |
| Adrenaline in Hospital | Administration of the adrenaline in emergency department (yes/no) | Time-dependent |
| Time to Adrenaline from Arrival | Time to administration the adrenaline from hospital arrival in emergency department (minute) |  |
| Intubation in Hospital | Intubation in emergency department (yes/no) | Time-dependent |
| Time to Intubation from Arrival | Time to perform the intubation from hospital arrival in emergency department (minute) |  |
| ECPR case volume | The hospitals are divided to three categories (High, middle, and low) based on the tertile of the number of cases ECPR performed in the recent 2 years. Low: 1~5 cases/2 years, middle: 6~14 cases/2years, High: more than 15 cases/2years | Fixed |
| Survival | 30-day survival | Outcome |
| Cerebral Performance Category (CPC) | Cerebral Performance Category: category 1, good cerebral performance; category 2, moderate cerebral disability; category 3, severe cerebral disability; category 4, coma or vegetative state; and category 5, death/brain death. [1] The CPC was evaluated by clinicians or research assistants in each hospital and registered to the database using the general instruction of CPC assessment of the study protocol. | Outcome |

We obtained the data for this analysis from the study committee in 2022. At that time, available and cleaned-up data ranged from 2014 to 2019.

ECPR, Extracorporeal cardiopulmonary resuscitation, CPR, Cardiopulmonary resuscitation, VF, Ventricular fibrillation, VT, Ventricular tachycardia, PEA: Pulseless electrical activity, ROSC, Return of spontaneous circulation, AED, Automated external defibrillator. *****Included in the model, fixed: We included the variable as the fixed covariates, time-dependent: We included the variable as the time-dependent covariates.

**S-Method 2. Missing imputation**

For dealing with missing variables, we used a machine-learning-based imputation technique to impute the missing values using the “missForest” package. [2, 3] This imputation technique is a nonparametric algorithm that can accommodate nonlinearities and interactions, and the single point estimates can be generated accurately by a random forest. [2, 3] The advantages of using the random forest model are that it can handle continuous as well as categorical responses, requires very little tuning and provides an internally cross-validated error estimate. [2, 3] This imputation technique is reported to be reliable and valid compared to the other imputation method such as k-nearest neighbors imputation or multivariate imputation using chained equations. [2, 3] Missingness was imputed using all predictors, outcomes, and other covariates.

**S-Method 3. Additional explanation for the method**

**Resuscitation time bias and risk-set matching with time-dependent propensity score**

Resuscitation time bias is a kind of bias in the observational study of resuscitation to investigate the association between a certain intervention and outcomes. [4] It is also well known as immortal time bias in the study of the critical care field. In terms of the association between ECPR and outcomes, the resuscitation time bias may occur if it is not appropriately dealt with because ECPR during cardiac arrest is related to the time course of the resuscitation. First, ECPR may be more likely to be implemented the longer the duration of the cardiac arrest, and generally, the longer duration of resuscitation is associated with a poor outcome. Second, once ROSC is obtained, ECPR can no longer be performed. Obviously, ROSC is linked to good outcomes. Consequently, if the analysis is performed without considering the time to implement the ECPR or the time to obtain the ROSC, it might wrongly indicate the contradictory result that ECPR is associated with poor outcomes. One way to deal with this resuscitation time bias is a risk set matching. In this method, a patient with ECPR is matched to a patient who has not yet received ECPR at the time that ECPR is started for the patient with ECPR. This approach is expected to eliminate resuscitation time bias for the intra-cardiac arrest intervention, provided the timing of the ECPR is accurate.

However, this risk set matching deals with resuscitation time bias, but not with traditional confounders including potential time-varying confounders. One of the methods for eliminating these confounding is time-dependent propensity score matching where the propensity score is calculated based on a Cox proportional hazards model which can include time-varying covariates. This study aimed to investigate the association between ECPR and clinical outcomes among OHCA patients using sequential risk-set matching analysis with a time-dependent propensity score to address resuscitation time bias.

**Exclusion criteria**

In our study, we intentionally excluded patients who were transferred to hospitals that were assumed to not have the capacity to provide ECPR. The exclusion criterion was based on the risk-set matching requirement that patients receiving exposure (ECPR) should be matched to the patients “at risk” of receiving exposure within the same minutes. In this case, "at risk" means patients who could potentially receive the ECPR but haven't. This condition ensures that there's a fair comparison between the treatment group and the control. Including patients transferred to hospitals without ECPR capabilities would mean these patients were not genuinely "at risk" of receiving the exposure. Therefore, by excluding such patients – who were directed to hospitals assumed to lack ECPR capabilities – we aimed to strengthen the fairness in comparison.

**Matching with replacements**

Matching with replacements of unexposed patients meant that matched controls with no ECPR at each time were allowed to match again later until they received ECPR. Therefore, at-risk patients also included those who received ECPR later, as matching was not dependent on future events. For example, assuming the situation that at the time point 20 minutes after hospital arrival, a patient treated with ECPR (patient A) and another patient treated without ECPR within this time (patient B) are matched. Patient B has the possibility to receive the ECPR at a later time (e.g., 25 minutes after hospital arrival) because future events are not decided at this time. Thus, the following situation can exist, Patient B received ECPR 25 minutes after hospital arrival and was matched to Patient C who did not receive ECPR within that time. As a result, Patient B is theoretically assigned to both groups (ECPR group and control group) in the matched cohort.

**S-Table 1. Missingness of the variables**

| Characteristic | Missing | |
| --- | --- | --- |
|  | Initial shockable rhythm  (n= 3,055) | Initial non-shockable rhythm (n= 30,231) |
| Sex | 0 (0%) | 0 (0%) |
| Age | 0 (0%) | 0 (0%) |
| Initial cardiac rhythm | 0 (0%) | 0 (0%) |
| Witness | 0 (0%) | 0 (0%) |
| Bystander CPR | 0 (0%) | 0 (0%) |
| Bystander AED | 0 (0%) | 0 (0%) |
| Physician Staffed Heli/Ambulance | 0 (0%) | 0 (0%) |
| Shock by Paramedic | 0 (0%) | 0 (0%) |
| Advanced Airway | 305 (10.0%) | 2,600 (8.6%) |
| Number of IV adrenaline | 1 (<0.1%) | 30 (<0.1%) |
| Prehospital ROSC | 0 (0%) | 0 (0%) |
| Time from Call to Hospital | 28 (0.9%) | 58 (0.2%) |
| Initial Cardiac Rhythm on Arrival | 0 (0%) | 0 (0%) |
| Shock in Hospital | 0 (0%) | 0 (0%) |
| Time to shock from Arrival | 454 (15%) | 671 (2.2%) |
| Adrenaline in Hospital | 22 (0.7%) | 322 (1.1%) |
| Time to Adrenaline from Arrival | 691 (23%) | 7,367 (24%) |
| Intubation in Hospital | 0 (0%) | 0 (0%) |
| Time to Intubation from Arrival | 672 (22%) | 5,931 (20%) |
| Censor Type | 0 (0%) | 0 (0%) |
| Time to Censor | 470 (15%) | 1,537 (5.1%) |
| 30-day Survival | 1 (<0.1%) | 1 (<0.1%) |
| 30-day Neurological outcome | 0 (0%) | 1 (<0.1%) |

ECPR, Extracorporeal cardiopulmonary resuscitation, CPR, Cardiopulmonary resuscitation, VF, Ventricular fibrillation, VT, Ventricular tachycardia, PEA: Pulseless electrical activity, ROSC, Return of spontaneous circulation, AED, Automated external defibrillator.

**S-Table 2. Disposition in the original cohort**

|  | Initial shockable rhythm | | Initial non-shockable rhythm | |
| --- | --- | --- | --- | --- |
| Characteristics | ECPR  (N=942) | Control  (N=2108) | ECPR  (N=376) | Control  (N=29855) |
| Censor Type |  |  |  |  |
| Death | 0 (0%) | 1,146 (54%) | 0 (0%) | 22,970 (77%) |
| ECPR | 942 (100%) | 3 (0.1%) | 376 (100%) | 1 (<0.1%) |
| ROSC | 0 (0%) | 959 (45%) | 0 (0%) | 6,884 (23%) |
| Time to ECPR | 26 (18, 36) | NA (NA, NA) | 28 (20, 39) | NA (NA, NA) |
| Survival (30-Day) | 231 (25%) | 314 (15%) | 44 (12%) | 355 (1.2%) |
| Favorable Neurological Outcome (30-Day) | 113 (12%) | 154 (7.3%) | 17 (4.5%) | 62 (0.2%) |

Continuous variables are median and interquartile range, and categorical variables are number and percentage (%). ECPR, Extracorporeal cardiopulmonary resuscitation, ROSC, Return of spontaneous circulation.

**S-Table 3. Disposition in the matched cohort**

|  | Initial shockable rhythm | | Initial non-shockable rhythm | |
| --- | --- | --- | --- | --- |
| Characteristics | ECPR (N=913) | Control (N=913) | ECPR (N=370) | Control (N=370) |
| Censor Type |  |  |  |  |
| Death | 0 (0%) | 405 (44%) | 0 (0%) | 263 (71%) |
| ECPR | 913 (100%) | 389 (43%) | 370 (100%) | 60 (16%) |
| ROSC | 0 (0%) | 119 (13%) | 0 (0%) | 47 (13%) |
| Time to ECPR | 25 (18, 34) | 36 (27, 46) | 28 (20, 39) | 47 (32, 52) |

Continuous variables are median and interquartile range, and categorical variables are number and percentage (%). ECPR, Extracorporeal cardiopulmonary resuscitation, ROSC, Return of spontaneous circulation.

**S-Table 4. Time of ECPR before matching**

ECPR group in the initial shockable (n=942)

| Timing (min) | Number of cases | Proportion (%) |
| --- | --- | --- |
| 0-9 | 20 | 2.12 |
| 10-19 | 266 | 28.24 |
| 20-29 | 283 | 30.04 |
| 30-39 | 200 | 21.23 |
| 40-49 | 101 | 10.72 |
| 50-59 | 25 | 2.65 |
| 60-69 | 16 | 1.7 |
| 70-79 | 18 | 1.91 |
| 80-89 | 5 | 0.53 |
| 90-99 | 4 | 0.42 |
| 100-109 | 2 | 0.21 |
| 110-119 | 2 | 0.21 |

Initial non-shockable (n=376)

| Timing (min) | Number of cases | Proportion (%) |
| --- | --- | --- |
| 0-9 | 5 | 1.33 |
| 10-19 | 87 | 23.14 |
| 20-29 | 109 | 28.99 |
| 30-39 | 81 | 21.54 |
| 40-49 | 47 | 12.5 |
| 50-59 | 27 | 7.18 |
| 60-69 | 7 | 1.86 |
| 70-79 | 5 | 1.33 |
| 80-89 | 6 | 1.6 |
| 90-99 | 1 | 0.27 |
| 100-109 | 1 | 0.27 |
| 110-119 | 0 | 0 |

**S-Table 5. Time of ECPR after matching**

In the initial shockable

|  | ECPR group (n=913) |  | Control group (n=913) |  |
| --- | --- | --- | --- | --- |
| Timing (min) | Number of cases | Proportion (%) | Number of cases | Proportion (%) |
| 0-9 | 20 | 2.19 | 0 | 0 |
| 10-19 | 266 | 29.13 | 33 | 8.53 |
| 20-29 | 283 | 31 | 76 | 19.64 |
| 30-39 | 186 | 20.37 | 126 | 32.56 |
| 40-49 | 91 | 9.97 | 77 | 19.9 |
| 50-59 | 21 | 2.3 | 27 | 6.98 |
| 60-69 | 16 | 1.75 | 17 | 4.39 |
| 70-79 | 18 | 1.97 | 14 | 3.62 |
| 80-89 | 5 | 0.55 | 9 | 2.33 |
| 90-99 | 4 | 0.44 | 4 | 1.03 |
| 100-109 | 2 | 0.22 | 0 | 0 |
| 110-119 | 1 | 0.11 | 4 | 1.03 |

Only the cases treated with ECPR are indicated.

In the initial non-shockable

|  | ECPR group (n=370) |  | Control group (n=370) |  |
| --- | --- | --- | --- | --- |
| Timing (min) | Number of cases | Proportion (%) | Number of cases | Proportion (%) |
| 0-9 | 5 | 1.35 | 0 | 0 |
| 10-19 | 87 | 23.51 | 3 | 5.26 |
| 20-29 | 107 | 28.92 | 8 | 14.04 |
| 30-39 | 81 | 21.89 | 8 | 14.04 |
| 40-49 | 43 | 11.62 | 15 | 26.32 |
| 50-59 | 27 | 7.3 | 17 | 29.82 |
| 60-69 | 7 | 1.89 | 1 | 1.75 |
| 70-79 | 5 | 1.35 | 2 | 3.51 |
| 80-89 | 6 | 1.62 | 3 | 5.26 |
| 90-99 | 1 | 0.27 | 0 | 0 |
| 100-109 | 1 | 0.27 | 0 | 0 |
| 110-119 | 5 | 1.35 | 0 | 0 |

Only the cases treated with ECPR are indicated.

**Reference**

1. Cummins RO, Chamberlain DA, Abramson NS, Allen M, Baskett PJ, Becker L, Bossaert L, Delooz HH, Dick WF, Eisenberg MS *et al*: **Recommended guidelines for uniform reporting of data from out-of-hospital cardiac arrest: the Utstein Style. A statement for health professionals from a task force of the American Heart Association, the European Resuscitation Council, the Heart and Stroke Foundation of Canada, and the Australian Resuscitation Council**. *Circulation* 1991, **84**(2):960-975.

2. Waljee AK, Mukherjee A, Singal AG, Zhang Y, Warren J, Balis U, Marrero J, Zhu J, Higgins PDR: **Comparison of imputation methods for missing laboratory data in medicine**. *BMJ Open* 2013, **3**(8):e002847.

3. Stekhoven DJ, Bühlmann P: **MissForest—non-parametric missing value imputation for mixed-type data**. *Bioinformatics* 2012, **28**(1):112-118.

4. Andersen LW, Grossestreuer AV, Donnino MW: **“Resuscitation time bias”—A unique challenge for observational cardiac arrest research**. *Resuscitation* 2018, **125**:79-82.
